# Is There Evidence of Health Risks From Exposure to Micro- and Nanoplastics in Foods?

**DOI:** 10.3389/fnut.2022.910094

**Published:** 2022-06-28

**Authors:** Elena Molina, Sara Benedé

**Affiliations:** Food Allergy Group, Department of Bioactivity and Food Analysis, Institute of Food Science Research (CIAL, CSIC-UAM), Spanish National Research Council, Madrid, Spain

**Keywords:** nanoplastics, microplastics, food contamination, human health, food allergy

## Abstract

The human health impact of exposure to micro (MP) and nanoplastics (NP) from food remains unknown. There are several gaps in knowledge that prevent a complete risk assessment of them. First, the fact that some plastics may be chemically harmful, either directly toxic themselves or because they absorb and carry other components, which makes these particles may possess 3 types of hazards, physical, chemical and biological. In addition, the levels at which toxic effects may occur are unknown and there is a lack of studies to estimate the levels to which we are exposed. Plastic particles can induce physical stress and damage, apoptosis, necrosis, inflammation, oxidative stress and immune responses, which could contribute to the development of diseases such as cancer, metabolic disorders, and neurodevelopmental conditions, among others. In addition, they may have effects on other pathologies that have not yet been studied, such as food allergy, where they could act modifying the digestibility of food allergens, increasing intestinal permeability, promoting an intestinal inflammatory environment or causing intestinal dysbiosis, which could promote food allergen sensitization. However, given the limited information on the presence of MP and especially NP in food, further research is needed to estimate whether they could amplify the risk of allergic sensitization to food proteins and to elucidate the risk to human health.

## Introduction

Plastics are now used extensively in our daily routine, with global production increasing rapidly from 1.5 million tons in 1950 to 348 million tons in 2017 (1). Due to the large production and the insufficient recycling, much of it ends up as plastic waste, reaching all environments and ecosystems. A recent study estimates that the ocean is polluted by 8.3 million plastics per cubic meter of water (2), posing a major environmental problem, especially because of their ease of dispersion and persistence (3). The origin of this pollution comes mainly (60–80%) from the degradation of accumulated plastic waste (4), releasing into our environment an extremely large number of small particles called microplastics (MP, 1–5,000 μm) and nanoplastics (NP, <100 nm) generated from the fragmentation and degradation of larger plastics depending on the type and environmental conditions to which they are exposed (5).

Once plastic debris has degraded, the MP and NP can be easily consumed by marine animals and, in this way, enter our food chain. However, plastic pollution is not only present in the sea. There are different sources of plastic particles (domestic, industrial, agricultural and fishing use/production/waste of products containing plastic particles) and different routes (mainly through water and air) by which they are released into the environment and eventually enter the food chain. It also reveals the potential spread of plastic particles through the environment and their impact on food products and beverages. In addition, MP and NP can also be generated from plastic food contact materials (6, 7).

It has been estimated that the human intake of MP can attain 66,000, 28,000 and 36,000 particles/day through fish, crustacean, and mollusk consumption, respectively (8), being these figures higher in countries such as Belgium, France or Spain, where shellfish consumption is high compared to countries like the UK (9). In addition, a recent study analyzed stool samples from 8 healthy volunteers, founding nine types of MP, with polypropylene and polystyrene being the most abundant (10).

High concentrations of plastic debris have been found in fish, crustaceans, and shellfish. Indeed, many of the 220 species founded to ingest microplastic in nature (such as mussels, oysters, clams, common shrimps, etc.) are of commercial importance for fisheries and aquaculture (11, 12). Apart from fish products, MP have also been found in honey, beer, sugar, soft drinks, sea salt, edible fruit and vegetables, and also water and milk, which highlights the impact of plastic-based packaging materials for MP contamination (10, 13). Furthermore, MP could also originate from filtration membranes used in dairy industries, as the material composition of the plastic particles found in the final product were similar to those used to manufacture filtration units (14). All cow's milk samples for adults and children recently analyzed contained MP, with an average of 40 particles/L (14). Special attention should be paid to dairy products used in infant feeding since polypropylene baby bottles, normally used to contain these products, release MP (values as high as 16,200,000 particles per liter) as consequence of sterilization treatment and exposure to high-temperature water, having estimated an average exposure to infants up to 12 months old of 14,600–4,550,000 particles per capita per day (6). All these estimates are subject to large amounts of variation; however, given methodological and data limitations, these values are likely underestimated. In the case of NP, the exposure could be higher, as their size allows them to pass through the intestinal epithelium (15). In fact, a few studies have identified NP in human food and beverages (16), and at the 2020 conference of the American Chemical Society, Dr. Halden's research group from Arizona State University presented the results of a study in which NP were detected, for the first time, in human tissues (17). These overwhelming figures indicate that environmental contamination with NP is a major factor to take into account in food safety's research field which leads us to the necessary and worrying question of how does the consumption of NP affect our health?

## Impact of Micro and Nanoplastics in Human Health

The impact on human health of plastic contamination of our food and beverages remains largely unknown. The presence of NP in food is already identified as an emerging risk in the European Union as it was described, in 2016, by the European Food Safety Authority (EFSA) (18). In 2017, EFSA announced five priority issues in the area of food safety, highlighting first and foremost, the presence of NP particles in food (19). However, these reports also highlighted the lack of scientific information available on this issue, and the need for further studies for a more comprehensive assessment. NP are of particular concern because, due to their size, they are prone to interact with biological systems. They can pass through the intestinal epithelium and then be transported to distant tissues, where they can cross cell membranes and stress cells (9). Studies focusing on the damage to and contamination level of animal species collected from the wild environment, or on the rate and biology of MP and NP uptake of animals fed with them in laboratory are increasing. However, and despite having evidence of MP and NP in foods and human tissues (16, 17), there is still scarce evidence available on the health impact of them from a normal diet.

The difficulty in evaluating human health effects of MP and NP in food lies in two main issues. First, the fact that some plastics may be chemically harmful, either directly toxic themselves or because they absorb and carry other components, also known as Trojan horse effects; and secondly the difficulty in separating the comparative exposure from pollution and via food and water. Regarding the first issue, it is still unclear whether the observed effects derive from the polymer itself or from a certain particle shape, size, or chemical composition. A recent study compared the effect of various polystyrene MP to non-plastic control particles with similar size and shape on *Daphnia magna* (20). Authors concluded that the observed sublethal effects were polymer-specific, related to the size and shape of the polymer, and do not result from particle exposure *per se*, revealing that MP toxicology is shape and size-dependent. Accordingly, Monikh et al. indicated that the toxicity potency of nanoscale plastic debris is influenced by their chemical compositions and size, concluding that data generated for the toxicity of one type of nanoscale plastic debris, may not be extrapolated to other types of plastic waste (21). Moreover, the fact that properties of synthetic mass-produced polymers of daily use are changing as a function of time spent in the environment, further complicates the risk assessment of these particles. Well-defined macropolymers considered safe upon production, over time can be transformed into a plurality of MP and NP that could be ill-defined (22).

**Figure 1:**
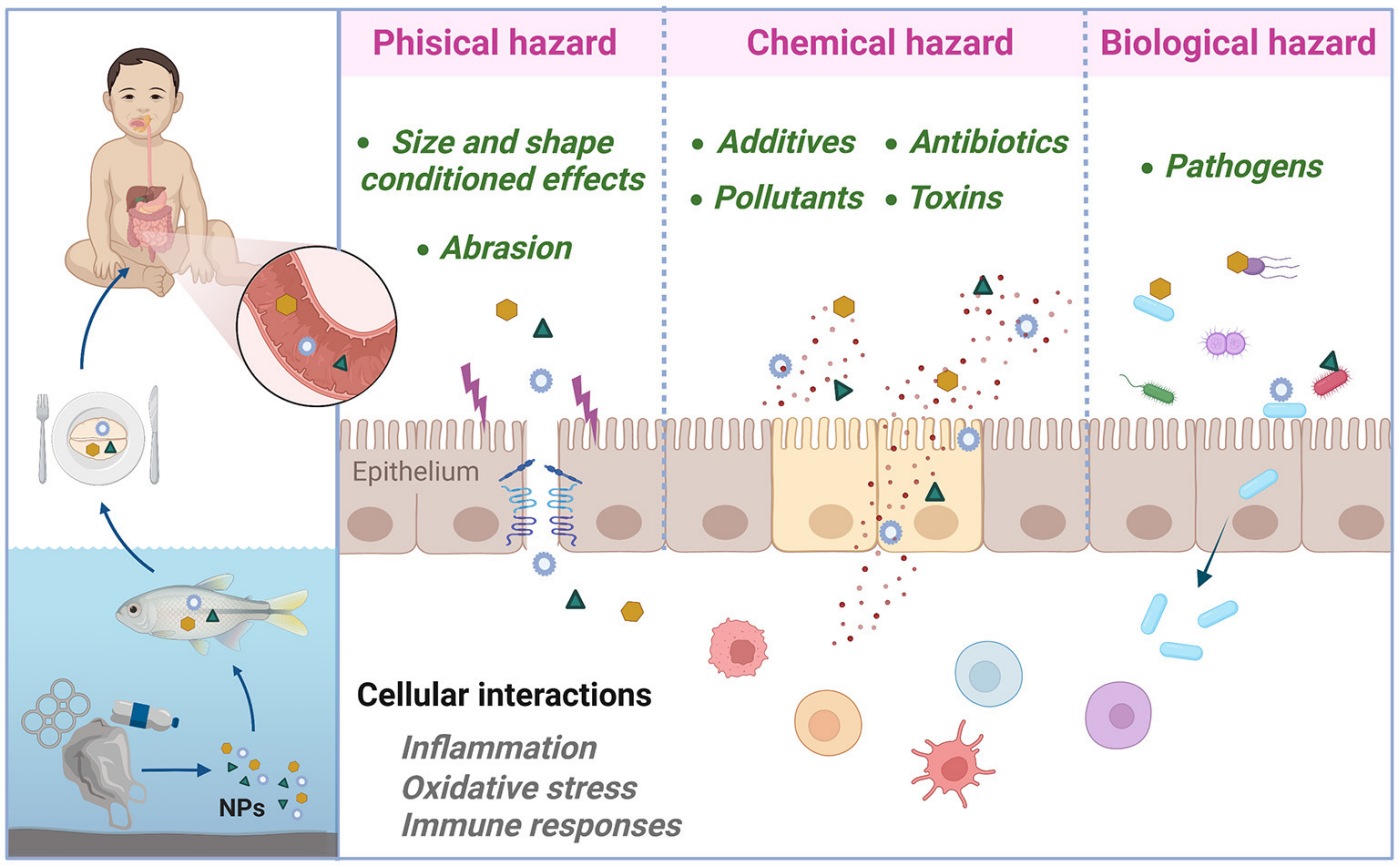
Potential mechanisms of nanoplastic (NP)-mediated food allergy sensitization and dysbiosis through the gastrointestinal tract. Created with BioRender.com.

It should also be considered that MP and NP can act as Trojan horse carriers, transporting 3 types of hazards, physical, chemical and biological (Figure 1). On the one hand, these plastic particles can cause physical injury by damaging the intestine when consumed with food, which is caused by the simple fact that plastics rub against tissue (23). Furthermore, plastic particles are not homogeneous; depending on the plastic material they come from, their size and also their shape, they will have different effects on our organism. For example, a recent study found significant differences in *Corbicula fluminea* depending if the organism was exposed to MP or NP derived from the same material. NP preferentially elicited the process related to cellular components and triggered the apoptosis through the mitochondrial pathway while MP induced the innate immune response and activated the complement system pathway (24).

Micro- and NP can also be a chemical hazard as they contain additives, which are added during their production to give them special properties such as strength, flexibility, stiffness, adaptability to external factors, etc. Food may contain additives as a consequence of the migration of these substances when it comes into contact with plastics (25), but in addition this migration may also come from plastic particles deposited on the food as a consequence of environmental contamination. Some of the most studied additives are phthalates and bisphenol A. Both are considered endocrine disruptors and can alter endocrine system functions leading to adverse developmental, reproductive, neurological, and immune effects, including abnormal growth patterns and neurodevelopmental delays in children (26). However, most of the studies to assess the health effects of phthalates and bisphenol A have been carried out on animals and limited scientific information exists on potential health problems in humans. Moreover, there are thousands of chemicals that consumers come into contact with every day, but most of them have not been studied, so the effects they or their mixture may have on human health are still unknown (27).

In addition, MP and NP have the ability to bind all kinds of compounds when they come into contact with fluids, thus acting as carriers of all kinds of substances (28). The type and amount of compounds they adsorb on their surface depends on the properties of the plastic (size, shape, composition, surface chemistry, etc.), the environmental conditions (contact time, temperature, pH, fluid composition, etc.) and the type of intermolecular forces generated between the various components of the fluid and the plastic particles (hydrophobic or electrostatic interactions, van der Waals forces, hydrogen bonds, etc.).

Substances that can be carried by MP and NP include numerous environmental pollutants such as polycyclic aromatic hydrocarbons, pesticides, chlorinated biphenyls or heavy metals such as cadmium, zinc, nickel and lead, among others (29, 30). These environmental pollutants, once adsorbed by the plastic particle, could be released when ingested and accumulate in the tissues, causing adverse effects on our health. Similarly, toxins produced by plants, bacteria, viruses and other micro-organisms or man-made processes could also reach our bodies and cause damage and diseases such as viral infections, gastrointestinal disorders, asthma, migraines or dermatitis, among others, and in the most serious cases, chronic pathologies such as heart disease or osteoarthritis, among others (31). In addition, antibiotics could also bind to the surface of MP and NP and, in the same way, be consumed through the diet, which could favor the appearance of antibiotic resistance, which has increased alarmingly in recent years, a fact that is currently of great concern to the scientific community (32).

In addition to environmental pollutants and biomolecules, MP and NP can attach living micro-organisms to their surface (33). The environment formed by the attachment of microbes to plastic waste is known as the plastisphere (34). The main problem with this phenomenon is that some pathogens can be found among the microorganisms attached to plastic, and therefore MP and NP can also pose a biological risk to human health. In addition, the chronic ingestion of MP and NP could impact on the natural community and abundance pattern of the gut microbiota which can result in triggering chronic diseases, promoting infections, and altering the gut microbiota (35).

Considering the above and the high variability of materials, shapes and sizes in which MP and NP can occur, the number of different potentially hazardous effects that these particles can cause is enormous and highlights the complexity of carrying out a risk assessment. In addition, given the variability of hazards they may pose, it is complex to estimate the exposure levels at which the harmful effects of each occur. Moreover, the amount of MP and NP present in food has been determine in very few foods and the obtained data are highly variable. Also, there are many foods we consume for which there are no available data, which complicates the determination of the amount of MP and NP we consume. In addition, there is a lack of studies examining the health impacts of MP and NP in human populations.

Although evidence exists on the relationships between some food system plastics and human health (particularly modeled exposures), there is insufficient comparable data to conduct meta-analyses around specific disease states (36). The results obtained from *in vitro* and *in vivo* studies reported that plastic particles can possibly accumulate and induce significant human health consequences, including physical stress and damage, apoptosis, necrosis, inflammation, oxidative stress and immune responses (37), which could be associated with the development of cancer (38, 39), metabolic disorders such as diabetes and obesity (40), neurodevelopmental conditions (41), reproductive problems, and asthma (42). In addition, they may have effects on other pathologies that have not yet been studied, such as food allergy, which has increased alarmingly in recent years in developed countries.

## Nanoplastics in Food Allergy

Environmental factors have been proven to contribute significantly to the development of allergic diseases, and environmental pollutants have also an impact in food allergy (43). Although their effect has been studied mainly in sensitization to aeroallergens, it seems that the implicated mechanisms could be, on the one hand, to damage the epithelium and therefore favoring the entry of the allergen into the body and the triggering of the immune response, and on the other hand, to act as transporters of allergens or adjuvants that modify the characteristics of allergens and increase the immune response to them. Environmental pollutants could influence the allergic sensitization process, not only through a respiratory route but also by an oral route, since many of them are present in foods and ingested daily, as it is the case of plastics.

Nanoplastics are of particular concern in this respect, because of their size they can be internalized and/or alter the biology of epithelial (44) and gut cells (45), leading to the idea that NP could promote allergic diseases. For example, there is a positive joint association between exposure in late pregnancy to chemicals that are used as plasticizers in plastic products and atopic dermatitis (46) and allergic airway inflammation (47). In addition, recent research has shown that oral exposure to bisphenol A exacerbates allergic inflammation in a mouse model of food allergy (48). Moreover, immune health is known to be closely linked to food allergies and intolerances, and it has been shown that MP in the gut can suppress the immune system and increase the likelihood of gastrointestinal diseases like inflammatory bowel disease (49). Additionally, although these effects have been related to exposure to MP, it is highly probable that NP cause even greater consequences on our health because, due to their characteristics and mainly due to their size, they could be able to enter our body through the intestinal barrier (15).

Against this background, and considering that NP contaminating food products may promote an inflammatory intestinal environment (50), it is not unreasonable to think that they could favor the development of allergic sensitization. In addition, the dysbiosis of the intestinal microbiome that NP may cause (51, 52), not only could have a direct impact on the intestinal health, but also could amplify the risk of food allergy. Several mechanisms could be involved in these processes.

Nanoplastics could potentially induce physical damages through particles themselves in the intestinal mucosa. The physical presence of NP may be toxic due to their inherent ability to induce intestinal blockage or tissue abrasion (53). The intestinal epithelium forms the first structural barrier against food allergens. The epithelial barrier function is mainly maintained by the formation of tight junctions, composed by zonula occludens 1–3, occludin and claudins 1–5, 20 and transepithelial allergen delivery could be facilitated by their disruption (54). In addition, as a reaction to stress or cellular injury, intestinal epithelial cells release alarming cytokines such as IL-33, IL-25, and TSLP, which expand group 2 innate lymphoid cells that produce IL-4, IL-5 and IL-13.30,31. These cytokines prime dendritic cells to induce a Th2 phenotype on T cells and also directly stimulate adaptive Th2 immunity and subsequent allergic reaction (55).

Nanoplastics could favor the intestinal absorption of allergens. Upon contact with human fluids, NP are immediately coated with proteins (56). Considering that the most abundant proteins are immediately adsorbed (57), and taking into account that food allergens are usually the most abundant proteins in food, the formation of an “allergenic protein corona” is highly probable in NP contaminated food during food processing or gastrointestinal digestion. The factors affecting this process include properties of NP, such as size, shape, composition, and surface chemistry; gastrointestinal conditions of NP, such as pH, time, and enzymes and food compound concentrations; and the interaction force between NP and proteins, such as hydrophobic and electrostatic interactions, van der Waals forces, and hydrogen bond interaction (58). The type of protein corona formed will determine the allergen interaction with cell membranes and the mechanism of cellular uptake. This could lead to an increased intestinal absorption of the allergen, favoring allergen sensitization, as an increased intestinal permeability has been observed in food allergic patients (59). In addition, food allergens present in the protein corona could be less accessible to digestive enzymes, reducing their digestibility and promoting sensitization, as it has been shown that protein must survive as intact protein or as large peptide fragments to be efficiently recognized by the immune system (60).

Nanoplastics could influence the gut microbiota. Recent evidence indicates that the susceptibility to food allergy is critically linked to microbial imbalance, also known as dysbiosis (61). Variations in gut microbiota following *in vivo* exposure to NP have been investigated in several contexts. All reports aiming to study the intestinal microbiota in NP exposed animals have observed dysbiosis. In the large yellow croaker fish, 14 days of exposure to polystyrene NP enhanced the relative abundance of the Firmicutes and the Bacteroidetes phyla, and diminished the abundance of the Proteobacteria (51), while in medaka, the abundance of Bacteroidetes increased (52). So far, the effect of NP in the microbiota of rodent models has not been evaluated, and even less in allergy mouse models; although, judging by the described background, it seems likely that NP exposure can also cause dysbiosis in these models, which could have an impact in allergic sensitization.

## Discussion and Conclusions

While the dramatic environmental impact of plastic waste rightly receives considerable attention from scientists, policy makers and the general public, the human health impact of plastic pollution in our food and beverages remains largely unknown. Moreover, in the absence of policies and mitigation strategies, it is likely to increase.

Realization of a conclusive human health risk assessment of MP and NP is currently not feasible because of the many gaps in knowledge we are faced (Table 1). The first step in risk assessment is hazard identification, which in the case of MP and NP is impaired because of the lack of effective, harmonized, and commonly available analytical methods tailored to the nano-range, as well as the limited availability of reference materials comparable to environmentally weathered plastics (5, 62). In addition, the spatial and temporal variability of MP and NP in the water, air or food is unknown which does not allow for standardized sampling protocols (63). The second step includes the hazard characterization which is hampered by the fact that plastic particles can present three types of hazards, physical, chemical and biological, which makes the identification and characterization of hazards very extensive and complex (23–32). Moreover, MP and NP are a multidimensional contaminant, differing in size, shape, polymer type, and additive cocktail, which could determine their toxicity (64, 65). In addition, there is limited available data on toxicokinetics and only include absorption and distribution, whereas no information is available on metabolism, interaction with microbiota or excretion, key processes in toxicity assessment (18). The third fundamental pillar in the risk assessment is the exposure assessment. Although there is evidence for the presence of MP and NPs in human tissues and feces, current scientific evidence on the exposure and toxicity of MP and NPs is very limited, and particularly little evidence remains available on the health impact of these materials following oral exposure through a normal diet. In this case, particular attention should be paid to NPs, as their size allows them to pass through the intestinal epithelium. Moreover, the levels at which toxic effects can occur are unknown and, although some of the sources and routes of exposure are well-known, it is difficult to estimate the levels to which we are exposed due to the lack of studies that determine the concentration of PM and NP in food (66). In addition, as MP and NP are present in food, they will be subjected to technological processing treatments during production or cooking and once ingested, to gastrointestinal digestion, which could alter the number and characteristics of the plastic particles that come into contact with the human body (67). On the other hand, most of the studies to assess the health effects of PM and NP have been carried out in animals and there is limited scientific information on potential health problems in humans and no epidemiological studies.

**Table 1:** Gaps in the knowledge and scientific uncertainties to perform a conclusive human health risk assessment of MP and NP exposure through food.

	**Topic**	**Gap in knowledge**	**Importance for risk assessment**	**References**
Hazard identification	Detection techniques	Lack of effective, harmonized, and commonly available analytical methods tailored to the nano-range	Necessary to identify the hazard	(5, 62)
	Standardization of analytical methods	Limited availability of reference materials comparable to environmentally weathered plastics	Important to establish comparability and accuracy of analytical results between different locations and over time	(5)
	Sampling	The spatial and temporal variability of MP and NP in a field is unknown	Necessary for standardizing sampling (how and how much to sample)	(63)
Hazard assessment	Hazard assessment	Many hazards posed by MP and NP are unknown and can be multiple (physical, chemical, and biological)	Identify the hazard posed by MP and NP is essential in order to carry out a complete risk assessment	(23–32)
	Toxicity	MP and NP are a multidimensional contaminant, differing in size, shape, polymer type, and additive cocktail. How these dimensions may influence the toxicity of the particles remains unknown. it is not yet possible to extrapolate data from one kind of particle to the other	Necessary to stablish toxic dose levels and evaluate acute and chronic toxicity	(64, 65)
	Fate of MPs and NPs in the human organism	There is a lack of knowledge about the impact of digestion on MP and NPs. The limited available data on toxicokinetics only include absorption and distribution, whereas no information is available on metabolism and excretion. Also, the local effects of MP and NP in the gastrointestinal tract, including microbiota, are unknown	Necessary to determine if ingested MP can be degraded to NP in the gastrointestinal tract. Moreover, toxicokinetic data are essential for a human risk assessment	(18)
Exposure assessment	Exposure levels	The routes and sources of exposure are not completely known	Necessary for estimating the human daily exposure	(66)
	Influence of food processing	Addition or removing of MP and NP from ingredients during processing or cooking and during the normal use of plastic food contact materials is unknown	It is crucial to estimate the contribution of food to overall human MP and NPs consumption and to mitigate this exposure in the future	(67)

Plastic particles can induce important epigenetic changes, including transgenerational effects (68), physical stress and damage, apoptosis, necrosis, inflammation, oxidative stress and immune responses, which could contribute to the development of diseases such as cancer, metabolic disorders and neurodevelopmental conditions, among others. In addition, they may have effects on other pathologies that have not yet been studied, such as food allergy, where they may act by modifying the digestibility of food allergens, increasing intestinal permeability to allergens, binding compounds with adjuvant activity, promoting an inflammatory gut environment and causing intestinal dysbiosis, which could promote sensitization to food allergens, contributing to the exponential increase in food allergies observed in recent years. However, given the limited information on the presence of MP and especially NP in food, further research is needed to assess their absorption in the organism or the effect of processing and to clarify whether plastic particles in food could amplify the risk of allergic sensitization to food proteins and to elucidate the risk posed by PM and NP to human health. Furthermore, the 2018 European Strategy for Plastics in a Circular Economy for the reduction of plastic use should be promoted, which will help to curb the generation of these particles and, consequently, their incorporation into food.

## Author Contributions

EM and SB compiled, researched, wrote, and edited the manuscript. Both authors approved the submitted version.

## Conflict of Interest

The authors declare that the research was conducted in the absence of any commercial or financial relationships that could be construed as a potential conflict of interest.

## Publisher's Note

All claims expressed in this article are solely those of the authors and do not necessarily represent those of their affiliated organizations, or those of the publisher, the editors and the reviewers. Any product that may be evaluated in this article, or claim that may be made by its manufacturer, is not guaranteed or endorsed by the publisher.
